# Cold-induced vasoconstriction for preventing onycholysis during cancer treatment

**DOI:** 10.1186/2046-7648-4-S1-A60

**Published:** 2015-09-14

**Authors:** Lola Bladt, Jonathan De Clercq, Tom Janssens, Johan Van Hulle, Jochen Vleugels, Jean-Marie Aerts, Guido De Bruyne

**Affiliations:** 1 Product Development, Faculty of Design Sciences, University of Antwerp, Belgium; 2 M3-BIORES, Faculty of Bioscience Engineering, KU Leuven, Belgium

## Introduction

Chemotherapy induced nail toxicity is observed in up to 88 % of cancer patients. Onycholysis, a severe form of nail toxicity in which the nail is detached from the nail bed, is observed in 0 % to 44 % of cancer patients undergoing a taxanes based chemotherapy. The use of ice gloves may reduce incidence rates for chemotherapy induced onycholysis, but cause cold and pain. In this research it was hypothesized that the use of local active cooling would reduce blood flow in the distal phalanxes, whilst inducing less discomfort as compared to an ice glove.

## Methods

Twelve healthy test persons, six male and six female, participated in this study. Average age was 22 years. Three test cases were induced (independent variables): active cooling of the right hand at 2 °C, active cooling of the right hand at 10 °C and passive cooling of the right hand with an ice glove cooled at -18°C prior to the test, resulting in 36 experiments. Active cooling controlled local skin temperature on the dorsal side of the proximal phalanges with the use of peltier elements. Local blood flow at the distal phalanges was assessed with the use of laser Doppler optometry (moorVMS-LDF2) under the nailbed and with the use of skin temperature (°C) measurements on the palmar side of the phalanges (dependent variable). Cooling effectiveness (%) was quantified as the relative change in the area under the curve of blood flow for distal phalanges I to V during the 30 minutes cooling period compared to a baseline that was measured for five minutes prior to the experiment. Ambient air temperature was 20°C (SE 0.4°). Thermal comfort was evaluated with the use of a likert scale.

## Results

This research showed that local active cooling of 2 °C is more effective (85.8 %, p < 0.05) compared to 10 °C (91.5 %) to induce vasoconstriction when blood flow is quantified through skin temperature. Blood flow assessed through laser Doppler optometry showed too large variations for providing conclusive results with respect to the effectiveness of passive cooling. Thermal comfort was significantly higher (p < 0.05) for local active cooling as compared to passive cooling.

## Discussion and conclusion

Local active cooling induces vasoconstriction at the distal phalanges of a hand and induces less discomfort as compared to an ice glove. More research is needed for providing insight in the underlying mechanisms of cold induced vasoconstriction and cold induced vasodilation to restrict blood flow under the nail bed and its effects on nail toxicity and onycholysis when taxanes are used during chemotherapy.

